# Relationships Between the Quantity and Quality of Pollen and the Quantity of Capped Brood in *Apis mellifera carnica* and *Apis mellifera caucasica*

**DOI:** 10.3390/ani15040611

**Published:** 2025-02-19

**Authors:** Krzysztof Młynek, Kalina Wnorowska, Grzegorz Pawluk

**Affiliations:** Institute of Animal Science and Fisheries, University of Siedlce, ul. B. Prusa 14, 08-110 Siedlce, Poland; krzysztof.mlynek@uws.edu.pl (K.M.);

**Keywords:** honeybees, subspecies, pollen, development, behavior

## Abstract

An important aspect in improving the efficiency of apiary production is knowledge of factors stimulating the condition of colonies. In view of climate change, one important factor is bee behavior associated with meteorological conditions. Studying the effect of selected meteorological parameters on honeybee colony development can help enhance management of the apiary economy. The aim of the study was to evaluate the connections between the quantity and quality of collected pollen and the quantity of capped brood, as an indicator of the rate of development against the background of weather condition indicators. We studied colonies of two relatively old subspecies: *Apis mellifera carnica* L. (CR) and *Apis mellifera caucasica* L. (CC) colonies. It was hypothesized that the quantity of pollen gathered is dependent on meteorological conditions, while the amount of capped brood depends on the quality and quantity of pollen. The study was carried out from April to July. Until May, both groups collected similar amounts of pollen. In the second part of the study, CC collected more pollen (averaging 32.3 g more). CR bees, however, exhibited greater development potential, with 6.32 g more capped brood on average (*p* ≤ 0.05). The greatest differences were noted in the spring period, which indicates faster development in CR colonies. The capped brood weight was positively correlated (0.391, *p* ≤ 0.05) with the amount of protein in the pollen. Trends in capped brood mass indicate that CR colonies were stronger during spring development. To some extent, this can be explained by the dependency of this behavior on meteorological conditions, specifically wind speed. In comparison to the CC subspecies (−0.600, *p* ≤ 0.05), CR bees were less sensitive to this factor, as indicated by the stronger correlation between pollen collection and wind strength (−0.642, *p* ≤ 0.05). The results indicate that some of the features of the subspecies we studied may have some potential in improving colony strength management in the context of changes related to the course of spring weather in eastern Poland.

## Simple Summary

Dynamic changes in climate increasingly result in atypical patterns of early spring and spring seasons. Insects such as honeybees are highly dependent on meteorological conditions during this period. Honeybees leave their hives, and the spring development of their colonies plays a crucial role in determining colony strength in subsequent seasons. Therefore, honeybee colonies must effectively adjust their behavior to adapt to spring ecosystem conditions. In this context, it is necessary to explore solutions that can support these beneficial insects. One such approach involves identifying honeybee strains with traits that enable colonies to adapt efficiently to the dynamics of changing spring meteorological conditions. This includes studying behaviors that ultimately promote colony development. In this study, particular focus was placed on analyzing the quantity and quality of collected pollen, as a source of protein, and the amount of capped brood, as an indicator of colony development. These indicators were examined in the context of meteorological conditions from April to July. Two subspecies were selected for the study: *Apis mellifera carnica* (CR) and *Apis mellifera caucasica* (CC). The results showed that CR exhibited higher developmental potential in spring, although CC collected more pollen later in the year. Meteorological conditions, particularly wind speed, significantly affected pollen collection. CR was more sensitive to this factor than CC. The usefulness of this study lies in expanding knowledge about the adaptive capacities of the investigated honeybee subspecies to dynamic springtime meteorological conditions. The findings may help optimize beekeeping practices in response to changing climatic conditions, particularly through the selection of honeybee subspecies adapted to specific ecosystems.

## 1. Introduction

The importance of honeybees as pollinators in natural habitats is increasingly weakened by climate change and human activities [1]. Honey yield is one of the most important traits in the performance evaluation of honeybee colonies. According to Thom et al. (2000) [2] and Robinson (2002) [3], it is determined by factors based in genotype–environment interactions. However, the primary factor affecting colony performance is the number of honeybees in good condition. In this context, diet—specifically access to pollen—plays a crucial role. Studies conducted by Frias et al. (2016) [4] indicate that limited pollen availability can disrupt brood rearing and reduce colony longevity. The amount of pollen collected by a colony is influenced by numerous factors [5]. Environment impeding behavior associated with gathering food for the colony’s development after wintering can indirectly destabilize the generational structure during the production season. This may be influenced by changes in biocenosis, which are strongly linked to climatic and geographical predictors [6]. This is especially important as, according to Toth and Robinson (2005) [7], interactions between groups of working bees also affects the propensity to gather food and the efficiency of processing nectar into reserves. This is particularly interesting in the context of the study by Ament et al. (2010) [8], which suggested that the behavioral maturation of honeybees is more closely related to their protein diet than to the growth and developmental phase associated with their age. This seems interesting in terms of possibilities of improving production efficiency, given that increasingly changeable meteorological conditions are observed during spring colony development. Studying the interactions between climate change and the ability of bees to adapt to it, especially during colony development, seems to be a particularly important area of research. This is mainly because pollen storage during this period is often limited by the number of eggs laid and the area occupied by the brood [9]. However, brood care behaviors can be subject to modification, especially when environmental conditions limit foraging and pollen collection [10]. They also influence the decision of the bees and extent to which it can be accumulated in cells [9,11]. Studies by Almeida-Muradian et al. (2005) [12] and Human and Nicolson (2006) [13] indicate that this is due to the colony treating pollen as the main source of protein and essential nutrients for brood development. The ability of bees to adapt to increasingly untypical weather in spring is of great cognitive importance—mainly because of the relationship between brood production in the colony and pollen storage, as indicated by the research presented by de Lima et al. (2016) [14].

According to Page and Fondrk (1995) [15], pollen collection is strongly influenced by the rate of development of the colony, which can limit the free space in which to accumulate it. Another important factor is pollen quality preferences and—a factor less well understood—the willingness of bees to fly on windy days [16,17,18]. All of this may influence the potential for efficient food accumulation for the developing colony. Assuming that capped brood is the highest ‘colony value’, this type of research seems to have important practical applications in terms of improving the efficiency of apiary production. This is especially important as climate change causes earlier plant growth in spring, but with longer dry periods, which largely affect the development and strength of the colony later on.

One of the most important differences between the honeybee subspecies used is the speed of colony development. This trait is particularly crucial during the spring development period, when the queen begins to lay eggs intensively. Another significant feature is the occurrence of brood-rearing pauses. These pauses result from the colony’s response to dearth periods. Such behaviors can influence both the efficiency of colony development and the utilization of available floral resources. Current research mainly focuses on the potential use of biological, morphological, and productive traits of different honeybee breeds in the context of rapidly changing climatic conditions [19,20]. The demand for high-performing honeybees and selection based on so-called productive traits has led to a reduction in the range of old breeds such as Caucasian (CC) and Carniolan (CR) bees [21,22]. The CR breed, due to its early spring development, is well-suited for early spring nectar sources [23]. Additionally, it is highly resistant to brood diseases. In contrast, the CC breed is known for its diligence and persistence in searching for food sources. However, unlike CR, CC colonies exhibit slow development in spring.

The traits of these breeds should be re-evaluated in the light of climate change, as the increasing dynamics of these changes affect the earlier blooming and flowering duration of plants, which, in turn, influences nectar abundance and pollen production [24]. Spring weather patterns significantly affect colony development and, consequently, the colony’s long-term performance. It is believed that the traits of the studied breeds may help beekeepers in Central and Eastern Europe adapt to climate change, especially in small-scale, sustainable beekeeping operations. In this context, the functional characteristics of old breeds remain important, despite being increasingly displaced from use for various reasons [25].

The aim of the study was to analyze the links between the quantity and quality of collected pollen and the amount of capped brood in *Apis mellifera carnica* L. (Carniolan subspecies) and *Apis mellifera caucasica* L. (Caucasian subspecies). It was hypothesized that the amount of capped brood is associated primarily with the quality of the pollen carried to the hive and the determination of foragers to collect nectar.

## 2. Material and Methods

### 2.1. Characterization of Honeybees

The study on honeybees (*Apis mellifera*) was conducted using ten colonies each from two subspecies: 10 hives with Carniolan (*Apis mellifera carnica* L., CR) and 10 hives with Caucasian (*Apis mellifera caucasica* L., CC). The queen bees of both studied breeds came from our own breeding and were sisters. The material for their rearing (larvae) was collected from colonies in which queens were bred in the pure breed (CR and CC). Each of the females (gyne) designated for the experiment was inseminated with semen of the same breed. In order to obtain age uniformity, the queens in each of the colonies selected for the study were replaced the season before. Each of the queens was given a unique number. This allowed them to be identified during the season and the experiment.

To eliminate error arising from the need for water, drinking troughs were placed behind the inner partition in mid-March. They were secured with a cushion filled with chaff, and the bees had access to them through a slit in the upper part of the partition. This allowed warm air to flow into the compartment with the trough and encouraged the bees to drink water. During flight periods, when the temperature exceeded 12 °C, stimulating feeding was applied to promote colony development. A syrup was prepared from edible sugar and water in a 1:1 ratio (INVERTIX 72 FAV, Diamant, Miejska Górka, Poland). The syrup was enriched with a complementary feed mixture (Promotor L47, LABORATORIOS CALIER, S.A., Barcelona, Spain). A dose of 5 mL of the preparation was used per 1 L of syrup. The bees also had access to water in external waterers located around the apiary. In spring, the bees had access to willows, hazel, and other trees including apple, plum, pear, and currant. A small part of their foraging area consisted of areas where meadow flowers were present.

The studied colonies were kept in 12-frame Wielkopolski-type hives equipped with hygienic low bottom boards. A sliding tray was installed beneath the mesh section of the bottom board. From February to the end of April, the tray was closed to increase the temperature inside the hive, which aimed to stimulate colony development.

The study was carried in 2021 from April to July in a commercial apiary in Poland (52°22′04.8″ N 22°44′01.6″ E). The hives were inspected on the 1st, 5th, 10th, 15th, 20th, and 25th day of each month. Measurements were made during the inspection: the area occupied by capped brood (the weight of brood covering the area was calculated), the amount of pollen brought to the hive, air temperature and humidity, and wind speed. During the inspection of the hives, the swarming mood of the bees was monitored. In order to avoid this behavior, the nests were loosened by adding empty combs.

### 2.2. Research Techniques

Capped brood weight (CBW) and the weight of collected pollen (WCP) were used to characterize the development of the colony. Capped brood weight (CBW) was estimated based on the number of cells per 1 cm^2^ of the comb surface on average. The surface occupied by the brood was outlined, and its area was measured on transparent polyester film using a planimeter. For this purpose, the surface area occupied by cells from CBW was determined. The surfaces were outlined on foil sheets applied to the combs. Then, we converted the number of cells per 1 cm^2^ into the obtained surface area and multiplied it by the average brood weight. The measurements were made in the year before the study, during routine honey collection. The measurement was taken for the first time in April, the second time was in June. The procedure for determining CBW included random measurement of larvae and pupae (from day 10 to 20 of development). The estimate of CBW was based on 100 randomly chosen capped brood (analytical weight). To better average CBW values, larvae and pupae were collected from 5 frames (10 individuals on each side of the honeycomb). They were collected from different parts of the capped brood.

The weight of collected pollen (WCP) was determined on the basis of the weight of the pollen pellets left by the bees on the pollen trap. The pollen was weighed on a laboratory scale with an accuracy of 0.5 g. (WTC 2000, Radwag, Radom, Poland). In order to obtain the average WCP value, the measurement was carried out for three days. Twice each month (beginning and end of the month). On sunny days, the collected pollen was placed on trays placed in the vicinity of the studied colonies. Bees willingly took the pollen to the hives (days when WCP was not measured). The proximate composition of the pollen was determined [26]. The content of dry matter (DW) was determined by the classical method using a moisture analyzer (MA 50.R, Radwag, Poland). Crude protein content was determined by Kjeldahl’s method using a conversion factor of N × 5.6 [27]. The collected pollen was dried (from each day separately). The dried pollen was ground (from each day separately) and then samples were taken for analysis. The results obtained from the annals were averaged. The content of crude ash was determined by combustion of the samples at 450 °C (muffle furnace FCF 26SH, CZYLOK, Poland). Fat content was determined by a modification of the Röse–Gottlieb method (2010). The weather indicators (wind speed, temperature, and humidity) were measured continuously. The tables present the results as averages of monthly measurements.

The meteorological indicators (wind speed, temperature, and humidity) were measured continuously from sunrise to sunset. Measurements were taken every two days (15 per month). The collected data were averaged for easier presentation. Measurements were made using a multi-parameter recorder (LB-757), cooperating with a thermometer sensor and a hygrometer (LB-715), and an anemometer (LB-746) (LAB-EL Laboratory Electronics s.j., Reguły, Poland. Probes measuring meteorological parameters were located approximately in the central part of the area where the study colonies were standing. In the characterization of meteorological conditions, the monthly average precipitation and the number of rainy days were also used. This information served as additional context for the analyzed parameters. The tables present the results as averages of monthly measurements.

### 2.3. Statistical Analysis

Statistical characteristics were calculated in Statistica 13.0 software (StatSoft Inc., Tulsa, OK, USA). Two-way analysis of variance (subspecies and month) was performed using a linear model (GLM) with repeated measures (4 hive inspections per month). The data were presented as least-squares means (LSM) and standard error of the mean (SEM). Means were compared using Duncan’s test at *p* ≤ 0.05. The observed trends in the distribution of selected variable values were visualized through graphical representations. These results were derived using multiple regression analysis employing the fitting of a polynomial function. The independent variable was determined prior to approximation of the model. The power of the model was determined using SEM and R^2^. Correlations between selected parameters were estimated using polynomial regression (*p* ≤ 0.05).

## 3. Results

Table 1 presents the parameters used to characterize the development of Carniolan (CR) and Caucasian (CC) honeybee colonies. The data indicate that in the first period of spring development (April), the efficiency of development of both subspecies was similar, as there were no statistically confirmed differences in April in either capped brood weight (CBW) or the amount of pollen collected (WCP).

Differences were noted, however, in May, when CBW was 8.8 g higher in the CR subspecies (*p* ≤ 0.05). Both subspecies, however, still exhibited similar determination in gathering pollen (on average 155.2 g) at this time. In June and July, the amount of capped brood stabilized, as indicated by its similar quantity in both subspecies (89.2 g on average). A different tendency was noted in the case of pollen gathering, as CC bees showed greater willingness to gather pollen in both months. In comparison to the CR subspecies, the differences in the amount of pollen collected were 7.8 g (*p* ≤ 0.05) in June and as much as 13.5 g (*p* ≤ 0.05) in July.

Data on the content of the major components of the pollen brought to the hive (Table 1) showed no differences indicating preferences of the subspecies for plants producing pollen with higher protein content. Pollen collected in June and July had the highest average protein content, at 24.1% DW. In comparison with this period, pollen from May and April contained 1.2% and 1.7% DW less protein, respectively (*p* ≤ 0.05). A similar tendency was observed for ash content, which was associated mainly with the month and not the preferences of the subspecies. Its content was highest in June (5.5% DW) and in July and May (averaging 5.1% DW), and lowest, by an average of 1.2% DW (*p* ≤ 0.05), in April. No differences were noted in the quality of the pollen brought to the hive by the subspecies of bees.

The average content of dry matter was highest in April and May, at 7.1% and 6.0%, respectively, and was similar in the pollen collected by both subspecies. It decreased in successive months of the study, ranging from 5.1% to 4.7% in June and July. The percentages of the pollen components were shown to be strongly correlated with capped brood weight, which was used to characterize the development of the colonies. Positive correlation coefficients were obtained for protein and ash content: 0.841 and 0.798, respectively (*p* ≤ 0.05). A negative value was obtained for the correlation with dry matter (−0.736, *p* ≤ 0.05).

The analysis of meteorological parameters (Table 2) showed that monthly temperatures during the study ranged from 12.5 °C in April (*p* ≤ 0.05) to an average of 24.2 °C in June and July (*p* ≤ 0.05). The temperature increased by 4.7 °C on average from April to May (*p* ≤ 0.05). The highest variation in this parameter (SD) was noted during this period, at 3.3 on average. The reverse tendency was observed for humidity, which was lowest in April and May—75.1% on average. In June and July, it increased on average by 4.3% (*p* ≤ 0.05).

An important parameter for the efficiency of forager bee flights is wind speed. It was highest in April and showed the highest variation in that month (SD = 3.9). Relatively light wind was noted in May and June, with an average speed of 7.7 m/s. Somewhat higher wind speed, on average by 3.2 m/s (*p* ≤ 0.05), was recorded again in July. Data on the relationships between meteorological conditions and CBW are in line with the expected tendencies. Air temperature was found to be most strongly correlated with this parameter, with a correlation coefficient of 0.899 (*p* ≤ 0.05). Positive, although much weaker, relationships were shown with humidity (0.447, *p* ≤ 0.05). A similar correlation strength was shown for wind speed, but it was negatively associated with CBW (−0.448, *p* ≤ 0.05).

The data in Table 3 show that the levels of pollen constituents were most influenced by air temperature. It was most strongly associated with protein and fat content: 0.738 and 0.663, respectively (*p* ≤ 0.05). The content of dry matter in the pollen, however, was negatively correlated with temperature (−0.656, *p* ≤ 0.05) and humidity (−0.548, *p* ≤ 0.05). The data in Table 3 indicate that meteorological conditions also had a strong influence on the amount of pollen collected (WCP). In general, the correlation values indicated relatively strong relationships between WCP and the analyzed meteorological parameters. The average values of the correlations were 0.705 for air temperature, 0.551 for humidity, and −0.608 for wind speed. However, the strength of the correlations depended on the subspecies of bee. The results show a tendency of weaker correlations in the case of CR bees.

**Figure 1:**
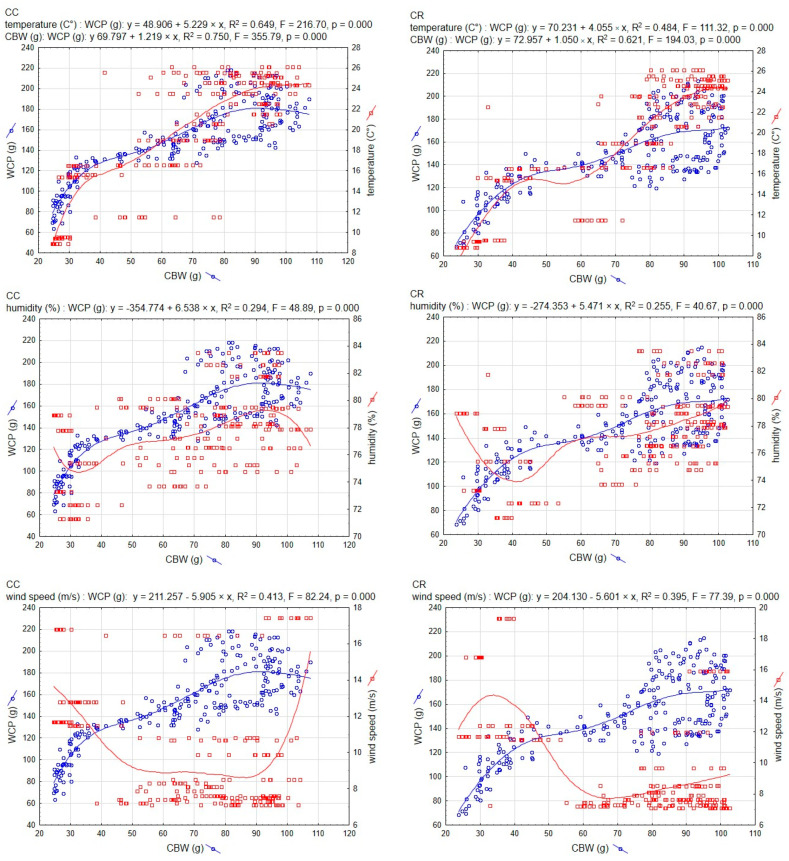
Multiple regression lines for correlations between the mass of capped brood—CBW and the mass of collected pollen—WCP (blue) and between CBW and meteo indices (red); CR—Carniolan, CC—Caucasian.

The results shown in Figure 1 illustrate the dynamics of the relationships observed between capped brood weight (CBW) and weight of collected pollen (WCP) in the honeybee subspecies we studied (CR and CC). Weather indicators assessed during the experiment served as a contextual background for analyzing colony development indices. The regression models for CBW and WCP in the studied subspecies exhibited a positive slope. The visualization of trends in CBW and WCP suggests that CC responded to increases in daily average values of these parameters with more active pollen collection. In the graphs showing results in relation to wind speed, a clear negative slope was observed for this weather parameter in relation to changes in CBW and WCP.

The R^2^ values presented in Figure 1 indicate that the regression models explained a substantial proportion of the variability in the studied indices. For the CBW–WCP relationship, the models accounted for variability at 62% (*p* = 0.000) for CR and 75% (*p* = 0.000) for CC. In contrast, the models assessing the relationship between pollen collection (WCP) and meteorological conditions explained variability most effectively in relation to air temperature. Values recorded for subspecies CC reached 64% (*p* = 0.000), while those for subspecies CR reached 48% (*p* = 0.000). Lower explanatory power was observed for the relationship between WCP and wind speed.

These values were 41% for subspecies CC (*p* = 0.000) and 39% for subspecies CR (*p* = 0.000). The models we employed explained the variability in the WCP–air humidity relationship to the least extent. In this case, the explained variance reached 29% and 25% for subspecies CC and CR, respectively (*p* = 0.000).

## 4. Discussion

Behavior associated with pollen gathering by honeybees (*Apis mellifera* L.) is a crucial feature of the development of their colonies. A study by Pankiw (2007) [28] showed that pollen is used to supply protein and primary source: fats, minerals, and vitamins. Pollen gathering is determined by numerous factors [28,29,30], but the overriding factor is the atavistic behavior of honeybees based on the instinctive search for food to meet the nutritional needs of the colony. The individual needs of the bees are secondary. Authors such as Dreller et al. (1999) [9] and Nelson and Jay (1972) [31] found that the rate at which pollen is brought to the hive is strongly associated with the development of the condition of the colony, especially when analyzed in terms of brood quality. This is due to better nutrition of the nurse bees, whose functional mandibular and hypopharyngeal glands can function more efficiently [32]. This also affects the condition of young larvae before the cells close [33,34]. Therefore, the nutritional quality of pollen is of great importance. Studies by Fewell and Winston (1992) [35] have shown that the willingness to collect pollen may be influenced by factors unrelated to its nutritional quality. In the study of these authors, a temporary state of strength of the colony was indicated. Importantly Clarke and Robert (2018) [36] showed that meteorological conditions also influence dust collection.

In the hypothesis proposed by Fluri et al. (1982) [37], it was suggested that bees emerging in the spring have less experience in pollen collection compared to those active in the summer. The patterns observed in our study regarding the development of WCP may also support this hypothesis, as the WCP values for the CR and CC subspecies studied were found to be very similar during the spring period (April, May). However, based on the CBW values obtained, it can be inferred that the CR subspecies may more effectively utilize the limited pollen available during this period. This finding is surprising; however, it is important to note that pollen only meets about 5% of the protein requirements for bees [38]. The majority of this nutritional requirement is supplemented by royal jelly, produced by nurse bees [39]. This ability in the CR subspecies could represent an important adaptation, potentially enhancing resilience to variable spring meteorological conditions. Notably, protein deficiency during larval development can impair growth and overall development, as reviewed by Brodschneider and Crailsheim (2010) [40]. It is also worth considering that the observed situation may be influenced by the energy (carbohydrate) content of royal jelly, which constitutes 18% to 45% of the larval diet, averaging around 59.4 mg [41].

In the mechanism whereby the nutritional value of pollen is transferred to the condition of the brood, an important role is played by a caste of workers known as nurse bees, whose task is to process the protein contained in pollen into protein-rich food used to feed the larvae and queen—e.g., royal jelly [42,43]. The division of labor in a bee colony model described by Breed et al. (1990) [44] can also have a significant impact in this regard. According to the authors, pollen reserves are dependent on the number of gatherers, which instinctively fly out to collect it. The studies cited to some extent explain the tendencies observed in our study in the strength of the relationships between the weight of collected pollen and the weight of capped brood.

Research conducted by Di Pasquale et al. (2016) [45] indicates that pollen consumption even 10% lower shortens the life of bees by up to three days. Although our research focuses on capped brood, it was also most strongly associated with the composition of the pollen; however, its mass (WSB) was related to the protein content in the pollen. In this case, the strength of this relationship was confirmed by the highest correlation coefficient.

According to DeGrandi-Hoffman et al. (2010) [42], an important factor determining the condition of bees is pollen reserves and an unlimited supply of protein. In their opinion, this is a crucial factor enhancing the immune responses of adult bees. They demonstrated that deformed wing virus (DWV) was more common in colonies fed exclusively on sugar syrup. This is most likely linked to the need for a sufficient supply of amino acids and energy to build body condition. This hypothesis may be confirmed by research by Yazlovytska et al. (2023) [46], which compared the survival of bees depending on their diet. The researchers observed that diets rich in pollen and beebread significantly improved bee longevity and elevated levels of key biomarkers associated with oxidative stress.

However, Kleinschmidt and Kondos (1976) [47] showed that the need for protein is also influenced by the amount of honey produced by the colony. The situation described by these authors appears to provide a logical explanation for the observed decline in brood weight (CBW) in the subspecies we have been studying since June. When kept under the same environmental conditions, both subspecies exhibited similar responses in this regard. It can be inferred that, in the colonies we studied (CC and CR), the nurse bees did not drastically reduce their interest in rearing young larvae. Nonetheless, both CC and CR showed an increased demand for pollen collection (WCP) during the summer season. This appears to be a reasonable explanation for the trends observed in our experiment, as the subspecies we studied were maintained under identical environmental conditions.

The trend we have shown is that in summer, a larger amount of capped brood is not necessarily accompanied by greater pollen collection. This discrepancy situation can also be explained by the quality of the pollen, in which the level of protein exceeded the 20% necessary for the development of the colony [47]. Based on our data, it can be concluded that during the spring development period, colonies of both subspecies adopted a similar strategy for pollen collection, gathering it with comparable intensity. However, at the beginning of summer, we observed higher pollen intake in CC colonies, while both subspecies showed similar values for capped brood weight. The differences in pollen collection observed in our study are partially consistent with the findings of Basualdo et al. (2000) [48], which demonstrated differences in pollen and nectar collection between honeybee subspecies. In light of this information, our results may also suggest that meteorological conditions play a significant role in shaping the foraging strategies of the studied subspecies. Specifically, the rationality behind pollen collection appears to be based on decisions associated with feeding the brood and the development of the colony. This is interesting in terms of the occurrence, during abundant nectar supply, of nurse bees neglecting larvae rearing. Our results may indicate that in comparison to the subspecies CR, CC workers more often decide to go and gather pollen, irrespective of the needs of the colony. However, according to Schneider and Hall (1997) [49], this is primarily the result of the flexibility in foraging behavior of different honeybee subspecies. Furthermore, Schneider and Hall (1997) [49] showed that pollen collection by different subspecies of *Apis mellifera* is more dependent on environmental factors than on genetic determinants. This is, of course, true; however, the bee subspecies we studied were maintained under the same environmental conditions and subjected to identical technical procedures. Therefore, in our opinion, the influence of genetic factors should not be categorically excluded.

On the other hand, however, the topic related to our results may indicate that in comparison to the subspecies CR, CC workers more often decide to go and gather pollen, irrespective of the needs of the colony. However, honey bee colonies are highly flexible in pollen collection, adjusting based on the amount of brood and the availability of pollen within the hive [50]. Our results correspond to those reported by Di Pasquale et al. (2016) [45], who observed the greatest demand for protein food in May and August. Moreover, we found that pollen collections were highest during the summer months. The greatest increase was noted at the end of April and the beginning of May. It is interesting that it was similar in both subspecies of bees. This situation may be explained by the flowering of rapeseed. Its intense blooming during this period could have heightened the bees’ interest in foraging. This observed trend was present in both subspecies we studied, suggesting that the instinct for food collection is strongly pronounced in both subspecies.

In contrast to these tendencies, however, studies by Page and Fondrk (1995) [15] indicate that the efficiency of pollen gathering depends on genetic factors. In our opinion, this is not a direct influence, and it is less pronounced during spring colony development for the breeds tested in our experiment development. We did observe greater capped brood weight in the Carniolan subspecies (CR) at that time, but its growth rate was similar to that of CC bees. This may indicate that CR bees have greater genetic potential in terms of stimulating the queens to produce spring broods and lay more eggs. This may be linked to the effect of meteorological conditions, which also influenced the pollen gathering tendencies observed in our study. Studies by Comba (1999) [51] and Vicens and Bosch (2000) [52] showed that wind may be one of the main factors reducing bees’ desire to gather food.

The tendencies observed in our study indicate that wind had a negative effect on the amount of pollen collected by both subspecies of bees. The values of this indicator obtained in our study were significantly higher than those reported by Czekońska et al. (2023) [53]. The authors noted that the bees they studied collected food when wind speed ranged between 2 and 4 m/s. However, the relationship they observed, which was also noted in our research, is consistent with the findings of Hennessy et al. (2020) [17], who found that wind speed negatively influenced the number of flowers visited. Our study also showed that the effect of the factors analyzed, including wind, was somewhat less strong in the Carniolan subspecies. We noted a similar tendency in the case of temperature. The results may indicate that Carniolan bees show greater determination to gather pollen at lower temperatures and higher wind speed. It is surprising that Czekońska et al. (2023) [53] reported a minimal impact of wind on changes in hive weight. However, this can be explained by the measurement of the entire hive’s mass rather than, as in our study, the analysis of the collected pollen mass. Additionally, other biocenotic factors likely contributed to the observed differences. According to cited authors, the change in behavior caused by stronger wind mainly involves hesitation before starting out. In our study, meteorological conditions were studied very generally. However, the results obtained are consistent with other reports, including the work of Burkle and Alarcón (2011) [54], which demonstrated that changes in weather patterns increasingly disrupt the synchrony between honey bee foraging and the phenology of *Apis mellifera* foraging sources. Unusual changes in the frequency of weather events or shifts in average meteorological conditions may reduce foraging activity [54] and alter flight patterns [55], which impairs foraging efficiency and weakens colony development. Although our study cannot definitively confirm such influences, the trends we observed may be partially explained by the findings of Karbassioon et al. (2023) [56] and Simioni et al. (2015) [57]. These studies showed that honey bee flight activity increases with temperature but is constrained by wind speed. Our results, however, may suggest that bees choose to forage at slightly higher wind speeds when air temperatures significantly exceed the minimum threshold tolerated by bees.

In the biocenosis conditions in which we conducted the research, meteorological conditions and the differences noted in the amount of pollen collected by the two subspecies of bees may be due to the potential of the subspecies in terms of courage to fly in less favorable weather. This particularly applies to the waiting time before taking off when the wind is strong. However, we did not find similar studies or data in the literature to confirm this hypothesis. Nonetheless, it should not be ruled out that, in the case of the subspecies we studied, wind speed could also have influenced foraging activity. In the biocenosis conditions in which this study was conducted, the obtained results may indicate a correlation between the behaviors related to the development of the colonies of the studied subspecies and meteorological conditions. In the weather indicators, the amount of pollen collected was shown to be associated with the colony’s needs, specifically in relation to the amount of brood being reared. A similar rate of increase in capped brood was shown in the two subspecies at the beginning of spring, but Carniolan bees were more willing to gather pollen. The rate of increase in brood weight was associated not only with the amount of pollen collected but also with its protein content. The amount of pollen collected was indirectly influenced by the determination of bees to fly when the wind was stronger and the temperature was lower. We believe that the obtained results are promising for practice but that further research is required in other biocenoses. Honeybee colonies typically survive for several years under relatively similar biocenotic conditions. However, the alignment of the colony’s developmental phenology may be constrained, particularly by weather fluctuations that affect the seasonal availability of blooming plants [58]. Therefore, monitoring the amount of collected pollen can help assess the impact of ongoing meteorological changes on foraging behavior, as well as the strength and growth rate of honeybee colonies.

## 5. Conclusions

Dynamic climate changes and the increasing instability of meteorological conditions are having a growing impact on the phenology of spring plants. Our research conducted in eastern Poland on two subspecies, *Apis mellifera carnica* (CR) and *Apis mellifera caucasica* (CC), has expanded knowledge about their adaptive capabilities and the influence of meteorological factors on pollen collection and colony development. During the spring, CR bees exhibited faster development, achieving higher amounts of sealed brood, which may indicate their superior growth potential during this period. In contrast, CC bees dominated in terms of pollen collection in the later months.

The data obtained indicate that the studied subspecies exhibit different flexibility in pollen foraging, especially under conditions of higher wind speed and temperatures significantly exceeding the bees’ minimum tolerance threshold. CR bees were found to be more sensitive to wind speed compared to CC bees. The study’s results can assist beekeepers in optimizing breeding practices by selecting honeybee subspecies best suited to local ecosystems. This knowledge not only improves apiary management efficiency but also helps better prepare for the challenges posed by a changing climate.

## Figures and Tables

**Table 1:** Parameters (x ± SD) characterizing the development of honeybee colonies, the quality of pollen brought to the hive, and their relationship with capped brood weight.

Parameter	Month (Number of Measurements)/Subspecies								Correlation x CBW
	April		May		June		July		
	CC	CR	CC	CR	CC	CR	CC	CR	
CBW (g)	29.9 ^d^	35.2 ^d^	65.6 ^c^	74.4 ^b^	87.5 ^a^	91.2 ^a^	90.9 ^a^	87.1 ^a^	-
	±11.6	±12.2	±5.1	±6.2	±5.4	±8.5	±8.3	±7.5	
WCP (g)	102.4 ^e^	109.5 ^e^	152.5 ^c^	157.9 ^c^	195.2 ^a^	187.4 ^b^	155.8 ^c^	142.3 ^d^	0.749 *
	±23.3	±28.4	±18.8	±23.1	±13.2	±14.7	±18.9	±16.5	
Pollen composition (% DW)									
Protein	22.5 ^c^	22.4 ^c^	22.9 ^b^	23.0 ^b^	23.9 ^a^	24.2 ^a^	24.6 ^a^	23.6 ^a^	0.841 *
	±0.8	±0.7	±1.1	±1.1	±0.9	±1.2	±0.9	±1.2	
Ash	3.9 ^c^	4.2 ^c^	4.9 ^b^	5.1 ^b^	5.5 ^a^	5.4 ^a^	5.1 ^b^	5.2 ^b^	0.798 *
	±0.5	±0.6	±0.5	±0.4	±0.2	±0.2	±0.4	±0.2	
Fat	6.5 ^b^	6.6 ^b^	6.7 ^a^	7.0 ^a^	6.6 ^a^	6.8 ^a^	6.5 ^b^	6.7 ^a^	0.248 *
	±0.2	±0.2	±0.4	±0.2	±0.5	±0.4	±0.4	±0.3	
DW	7.1 ^a^	7.1 ^a^	6.2 ^b^	5.8 ^b^	4.8 ^c^	4.7 ^c^	4.9 ^c^	5.1 ^c^	−0.736 *
	±1.4	±1.1	±1.0	±0.3	±0.4	±0.6	±1.1	±1.2	

^a,b,c,d,e^—*p* ≤ 0.05, * *p* ≤ 0.05, honeybee subspecies: CR—Carniolan, CC—Caucasian, CBW—capped brood weight, WCP—weight of collected pollen, % DW—content in dry weight.

**Table 2:** Means (x ± SD) for parameters characterizing meteorological conditions during the study and their effect on capped brood weight (CBW).

Parameter	Month (Number of Measurements)				Correlation CBW (g) x
	April (15)	May (15)	June (15)	July (15)	
Temperature (°C)	12.5 ^c^ ± 3.2	17.2 ^b^ ± 3.1	23.9 ^a^ ± 1.5	24.5 ^a^ ± 1.1	0.899 *
Humidity (%)	74.8 ^b^ ± 2.4	75.4 ^b^ ± 2.3	80.2 ^a^ ± 2.2	78.6 ^a^ ± 1.6	0.447 *
Wind speed (m/s)	13.4 ^a^ ± 4.3	7.6 ^c^ ± 2.4	7.8 ^c^ ± 3.1	10.9 ^b^ ± 3.9	−0.448 *

^a,b,c^—*p* ≤ 0.05, * *p* ≤ 0.05.

**Table 3:** Relationships between meteorological parameters and the content of constituents of pollen collected and its quantity (WCP).

Correlation (Significance *p* ≤ 0.05)	Analyzed Pollen Components (% DW)				Correlation Within Group WCP (g) x	
	Protein	Ash	Fat	Dry Weight	CC	CR
Temperature (°C)	0.738 *	0.442 *	0.663 *	−0.656 *	0.721 *	0.688 *
Humidity (%)	0.125	0.267	0.241	−0.548 *	0.554 *	0.548 *
Wind speed (m/s)	0.234	−0.456 *	0.134	−0.301	−0.642 *	−0.574 *

* *p* ≤ 0.05, WCP—weight of collected pollen, honeybee subspecies: CR—Carniolan, CC—Caucasian.

## Data Availability

The results of the research and information on the solutions used in the experiment are available to the authors of the manuscript. They have been collected in the form of a database.
